# Editorial: Analysis of tumor immune microenvironments and molecular mechanism to reveal the dilemma of immunotherapy for advanced non-small cell lung cancer

**DOI:** 10.3389/fimmu.2024.1415608

**Published:** 2024-04-25

**Authors:** Giandomenico Roviello, Ismaela Anna Vascotto, Martina Catalano

**Affiliations:** ^1^ Department of Health Sciences, Section of Clinical Pharmacology and Oncology, University of Florence, Florence, Italy; ^2^ School of Human Health Sciences, University of Florence, Florence, Italy

**Keywords:** non small cell lung carcinoma (NSCL), immunotherapy, biomarkers, chemotherapy combination therapy, predictive factor

Over the past two decades, immunotherapy has revolutionized cancer treatment, yielding unprecedented advancements in managing various advanced and metastatic malignancies. Immune checkpoint inhibitors (ICIs), represent pivotal tools approved for the treatment of numerous tumors, either alone or in combination (1). However, despite their efficacy, these therapies are hindered by limitations such as low response rates and the development of resistance mechanisms, which can compromise treatment outcomes and occasionally lead to severe side effects (2). Consequently, researchers have extensively explored strategies to augment immunotherapy’s efficacy, including its combination with conventional chemo/radiotherapy.

This Research Topic focuses on the analysis of immune TME and molecular mechanisms, discovery of novel cancer biomarkers and mechanisms to overcome the tumor’s immune resistance in advanced non-small cell lung cancer (NSCLC).

In their study, Chen et al. discussed the changes in the TME in NSCLC following treatment with tyrosine kinase inhibitors (TKIs). They highlighted that different oncogenic driver mutations, such as EGFR, KRAS, and MET, can result in varying TME compositions. Specifically, tumors with KRAS and MET mutations tend to have more CD8+ T cell infiltration compared to EGFR-mutated tumors.

The study suggests that responsive TKI treatments can remodel the TME by increasing immune-activated components like tumor-infiltrating immune cells and upregulating their function, while decreasing immunosuppressive components. The TME changes post-TKI treatment may be dynamic and dependent on factors like treatment period, sensitivity, and regimen of TKIs.

Regarding novel investigated biomarker, Li et al. observing that Integrin alpha-8 (ITGA8) protein expression levels were lower in lung adenocarcinoma (LUAD) and lung squamous carcinoma (LUSC) compared to normal tissues. Notably, ITGA8 expression was diminished in LUAD cell lines compared to human bronchial epithelial cell lines. Furthermore, survival analysis revealed a positive correlation between high ITGA8 expression in LUAD patients and improved disease specific survival, progression-free survival (PFS), and overall survival (OS), suggesting a potential tumor suppressive role for ITGA8 and its association with favorable prognosis in LUAD. Consequently, ITGA8 emerged as a promising candidate for diagnostic, therapeutic, and prognostic applications in LUAD.

Likely, Gao et al. study, authors explored the expression of proprotein convertase subtilisin/kexin type 9 (PCSK9) in tumor tissue from advanced NSCLC patients prior to receiving programmed death/ligand-1 (PD-L1) immunotherapy. Their findings revealed that high PCSK9 expression in baseline NSCLC tissues not only correlated with poor treatment response but also emerged as an independent risk factor for both PFS and OS in patients receiving anti-PD-1.

Regarding potential therapeutic implications, PCSK9 inhibition was found to enhance the antitumor effect of anti-PD-1 treatment *in vivo* and could potentially synergize with CD137 co-stimulation to further augment T-cell activation. This combination therapy demonstrated promising results in syngeneic mouse models, delaying tumor growth and promoting long-term survival.

The Hirayama et al. study highlighted the significant role of the inflammatory cytokine IL-1β within the TME of NSCLC, specifically in the upregulation of PD-L1 expression in tumor cells. Through a comprehensive analysis of literature, authors identified macrophages as the primary source of IL-1β in the NSCLC TME. Furthermore, they observed an increase in the IL-1β gene expression within myeloid cells in tumor tissue compared to normal lung tissue, which correlated with disease progression.

Their findings revealed that IL-1β alone induces the upregulation of PD-L1 expression in certain NSCLC cell lines and inhibits lymphocyte-mediated cell lysis suggesting a potential therapeutic strategy to inhibit PD-L1 expression and enhance immune-mediated tumor elimination.

Shimizu et al. conducted an individual patient data meta-analysis to evaluate the prognostic significance of soluble PD-1 (sPD-L1) changes during treatment with PD-1 inhibitors advanced NSCLC patients. A previous meta-analysis suggested that elevated pre-treatment sPD-L1 levels were associated with shorter OS and PFS (3). However, the relationship between sPD-L1 changes during treatment and prognosis has been inconsistent in previous studies, possibly due to small sample sizes or unclear clinical implications.

Recent research by Himuro et al. indicated a negative correlation between sPD-L1 changes during ICI therapy and OS in advanced NSCLC patients (4). Contrariwise, this analysis did not find a significant association between sPD-L1 changes and OS during PD-1 inhibitor therapy. This suggests that while sPD-L1 changes may not predict outcomes for PD-1 inhibitor treatment, they may still hold predictive value for PD-L1 inhibitor treatment.

The Li et al., research focuses on identifying cerebrospinal fluid (CSF) biomarkers for leptomeningeal metastasis (LM) in LUAD patients. In their previous study authors identified CEACAM6 as a potential biomarker for LM, but it lacked validation in LUAD patients without LM.

In this study, their enrolled patients without LM to further validate CEACAM6’s diagnostic efficacy for LUAD LM patients. Findings confirmed the high diagnostic accuracy of CSF CEACAM6, surpassing previous reports. They also observed that CSF circulating tumor cells influenced CSF CEACAM6 levels. However, CSF CEACAM6 quantification is not clinically approved but efforts are underway to establish a suitable method for clinical detection.

Recent advancements in immunotherapy have enhanced radical resectability in locally advanced NSCLC by improving downstaging (5). This has led to increased interest in combining chemotherapy with immunotherapy for conversion therapy in stage IIIC NSCLC. In their study Fu et al. reported a case of conversion therapy with immunotherapy and chemotherapy in an initial inoperable stage III NSCLC patient.

The patient achieved pathological complete response and experienced a favorable PFS after surgery. This conversion strategy holds promise for a subset of NSCLC patients, potentially enabling them to undergo radical surgery.

The findings discussed in these studies shed light on various aspects of immunotherapy in NSCLC, including the analysis of the TME dynamics, the identification of novel biomarkers, and the exploration of inflammatory cytokines in modulating PD-L1 expression and immune response. Additionally, the evaluation of sPD-L1 changes during treatment and the identification of CSF biomarkers for LM provide valuable insights into prognostic markers and diagnostic tools for NSCLC. Moreover, the reported case of conversion therapy in an initially inoperable NSCLC patient highlights the potential of innovative treatment strategies to improve patient outcomes.

Overall, the findings presented in these studies underscore the importance of personalized approaches and continued research efforts to overcome the challenges associated with immunotherapy in NSCLC.

## Author contributions

GR: Conceptualization, Data curation, Supervision, Validation, Visualization, Writing – original draft. IAV: Investigation, Writing – original draft. MC: Data curation, Supervision, Validation, Writing – original draft.
